# Angulated Needle Phlebectomy: A Novel Technique for Removing Small Reticular Veins

**DOI:** 10.1111/jocd.70694

**Published:** 2026-01-29

**Authors:** Wei‐Ming Wu, Ling‐Yi Wu

**Affiliations:** ^1^ DR WU Skin Clinic Kaohsiung Taiwan; ^2^ California Institute of Technology Pasadena California USA

To the Editor,

Small, unsightly reticular veins are a frequent concern in dermatology clinics. While standard treatments like sclerotherapy are common, they can be complicated by extravasation, long‐term hyperpigmentation, and the risk of arterial injection [1]. Furthermore, light‐based therapies are often not effective for vessels larger than telangiectasias. We have developed a novel surgical method, which we term *angulated needle phlebectomy*, which uses a modified hypodermic needle to hook the desired vessels.

The technique is as follows: After marking the target veins, multiple punctures are made with an 18G needle. A 23G needle is then bent at its tip to an angle of 90 degrees or more with the use of a needle holder. This “angulated needle” is inserted through a puncture and advanced along the vessel. The needle is then rotated 5–7 times to grab the vein before pulling it out, extracting the vessel (Figure 1).

**FIGURE 1:**
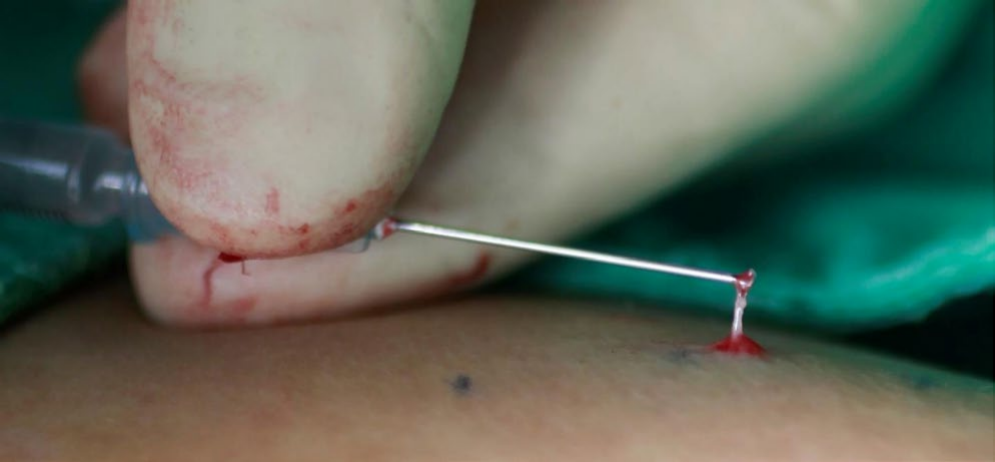
A small reticular vein hooked with the angulated needle.

Figure 2 shows two photographs of a representative patient in which the targeted vein was removed, before and 1 week after the procedure. Notably, the procedure left only transient ecchymosis and tiny erythema from needle wounds. There was no observable scar or linear hyperpigmentation, a common side effect from thrombosed veins in sclerotherapy.

**FIGURE 2:**
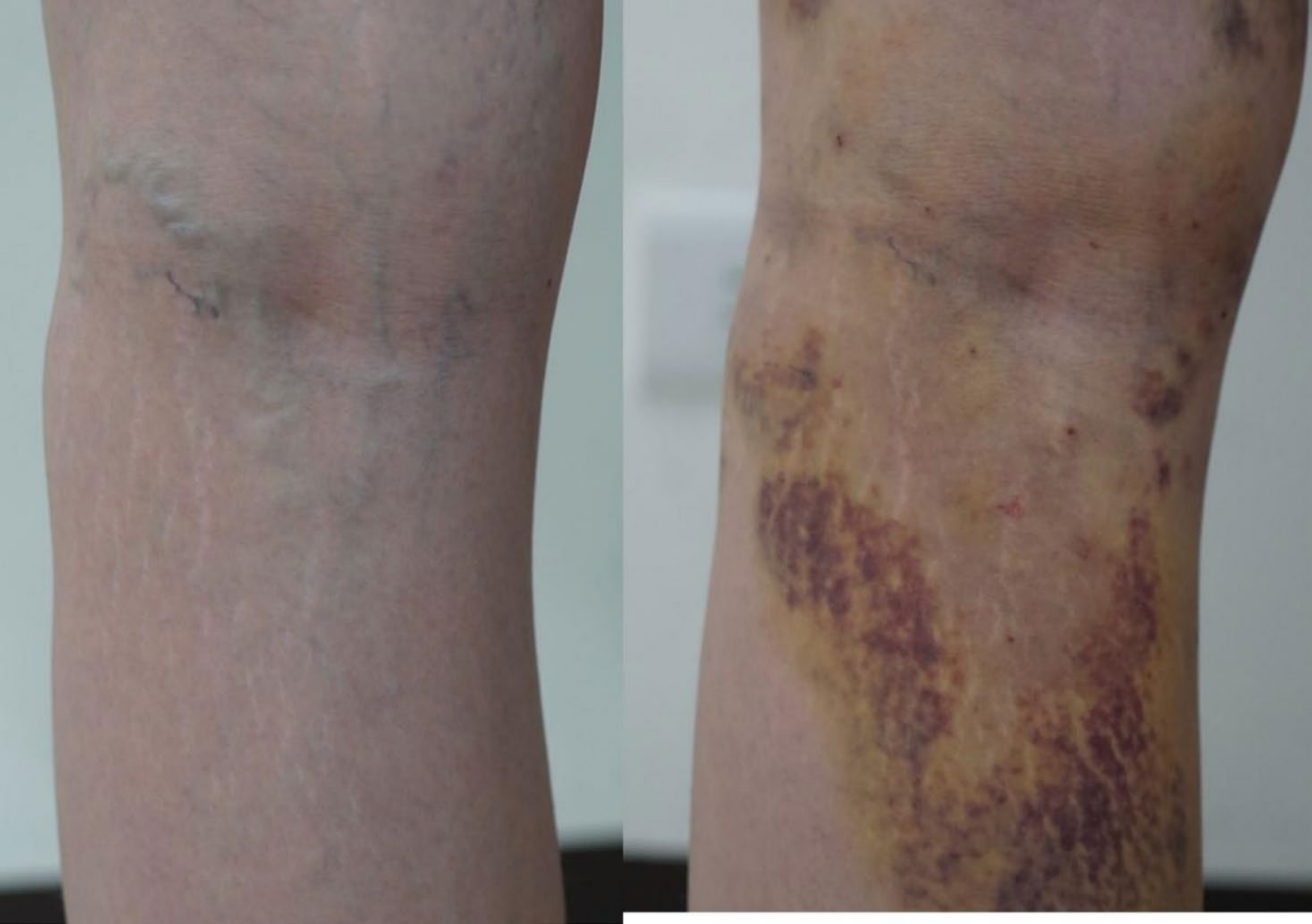
The photographs before and 1 week after the procedure. The target veins were removed and left surrounding ecchymosis.

The technique differs from traditional hook phlebectomy [2] in its usage of the sharp tip of the 23G needle: It pierces the vascular wall multiple times in advancing and rotating parallel to the vessel, providing a stronger grasp than simple vertical hooking. Smaller vessels (0.1–0.5 cm in diameter), often too small and fragile to hook in our practice, become accessible with this technique. The method also eliminates the need for precise intravascular placement and the risk of extravasation and intra‐arterial injection associated with sclerotherapy.

In clinical practice, this method also greatly facilitates the clearance of residual avulsed veins during hook phlebectomy because of its stronger grasping effect. Due to its small size and limited mechanical hooking strength, the angulated needle technique is not intended for the treatment of larger varicose veins; however, it may be particularly helpful for small reticular veins that are often difficult to engage with standard hook phlebectomy. Its use may also be limited for very small telangiectasias, but removal of the primary feeding reticular vein can still achieve satisfactory cosmetic results. In our clinics, we have used this technique either as a standalone procedure or as an adjunct to hook phlebectomy for small reticular veins, predominantly involving the lower extremities, including the thighs and calves, in several dozen patients. Follow‐up ranged from 1 to 4 months. The side effects observed were limited to transient ecchymosis and mild erythema at the needle entry sites, which typically resolved more rapidly than after sclerotherapy, likely due to the absence of residual damaged or thrombosed vessels. No cases of infection, scarring, nerve injury, or persistent hyperpigmentation were observed. This simple, minimally invasive technique can be readily applied in dermatology clinics and serves as a valuable addition to the treatment armamentarium for varicose and reticular veins.

## Author Contributions

**Wei‐Ming Wu:** conceptualization, methodology, formal analysis, writing – original. **Ling‐Yi Wu:** data curation and writing – review and edit.

## Ethics Statement

This study was conducted in accordance with the principles of the Declaration of Helsinki. All participants provided written informed consent in the study.

## Conflicts of Interest

The authors declare no conflicts of interest.

## Data Availability

The data that support the findings of this study are available on request from the corresponding author. The data are not publicly available due to privacy or ethical restrictions.
